# The distinct expression patterns of claudin-10, -14, -17 and E-cadherin between adjacent non-neoplastic tissues and gastric cancer tissues

**DOI:** 10.1186/1746-1596-8-205

**Published:** 2013-12-10

**Authors:** Man Gao, Wei Li, Haiming Wang, Guanjun Wang

**Affiliations:** 1 Cancer Center, the First Hospital of Jilin University, Changchun, Jilin, China; 2 Urinary surgery department, the First Hospital of Jilin University, Changchun, Jilin, China

**Keywords:** Gastric cancer, Cell adhesion, Tight junction, Claudin-10, Claudin-14, Claudin-17, E-cadherin, Immunohistochemistry

## Abstract

**Background:**

Recent data indicate that the cell adhesion proteins are abnormally regulated in several human cancers and the expression of the cell adhesion proteins E-cadherin and claudin proteins is involved in the etiology and progression of cancer. It is clear that these protein represent promising targets for cancer detection, diagnosis, and therapy.

**Methods:**

To explore the expression distinction of the cell adhesion proteins claudin-10,-14,-17 and E-cadherin in the adjacent non-neoplastic tissues and gastric cancer tissues, 50 gastric cancer tissues and 50 samples of adjacent non-neoplastic tissues adjacent to the tumors were examined for expression of claudin-10,-14,-17 and E-cadherin by streptavidin-perosidase immunohistochemical staining method.

**Results:**

The positive expression rates of E-cadherin in gastric cancer tissues and adjacent non-neoplastic tissues were 32% and 74% respectively (*P* < 0.01). The positive expression rates of claudin-10 in gastric cancer tissues and adjacent non-neoplastic tissues were 24% and 72% respectively (*P* < 0.01). The positive expression rates of claudin-17 in gastric cancer tissues and adjacent non-neoplastic tissues were 18% and 70% (*P* < 0.01). In contrast, the positive expression rates of claudin-14 in gastric cancer tissues and adjacent non-neoplastic tissues were 58% and 24% respectively (*P* = 0.015 < 0.05) Thus in our study, the expression of E-cadherin, claudin-10, and claudin-17 was down-regulated in gastric cancer tissue while the expression of claudin-14 was up-regulated. Correlations between claudins and E-cadherin expression with lymphatic metastasis were observed.

**Conclusion:**

Our study reveals that the expression of E-cadherin, claudin-10, and claudin-17 were down-regulated in gastric cancer tissue while the expression of claudin-14 was up-regulated and correlation between claudins and E-cadherin expression with lymphatic metastasis were observed.

**Virtual slides:**

The virtual slide(s) for this article can be found here: http://www.diagnosticpathology.diagnomx.eu/vs/1475928069111326.

## Introduction

Cell-cell adhesion junction is an essential necessity during cell differentiation, tissue development, and tissue homeostasis [1]. It is reported that the development of malignant tumors, in particular the transition from benign lesions to invasive, metastatic cancer, is characterized by a tumor cell’s ability to overcome cell-cell adhesion and to invade surrounding tissue and loss of cell-cell adhesion is displayed in many cancers and correlates with metastasis and poor prognosis [2]. Many different cell-adhesion molecules are implicated in human carcinogenesis [3]. During the transition from normal cells to highly malignant tumor cells, the expression of some of these adhesion molecules is switched off, whereas that of others is induced. E-cadherin, a transmembrane adhesion protein, is the major constituents of the epithelial cell junction system [4]. Besides E-cadherin exerts a potent invasion-suppressing role in tumor cell lines and in in vivo tumor model systems and loss of E-cadherin expression during tumor progression has been observed in numerous human carcinomas [5]. It is revealed that DNA hypermethylation and chromatin rearrangements within the regulatory regions of the E-cadherin gene have been correlated with the loss of E-cadherin expression in primary hepatocellular and breast carcinomas [6-8]. Other observations suggest that the loss of E-cadherin transcription in cancer cells is primarily due to transacting pathways regulating E-cadherin gene expression [9,10]. Besides supporting cell-cell adhesion, E-cadherin can affect a wide range of cellular functions that include activation of cell signaling pathways, regulation of the cytoskeleton and control of cell polarity [11-13].

Tight junctions, together with adherent junctions and desmosomes, form the apical junction complex in epithelial and endothelial cellular sheets [14-16]. The claudin protein family have a crucial role in formation of tight junctions (TJs), and consists of approximately 27 members, which are expressed with a tissue-specific distribution [17]. Because of the ability of tight junction proteins to recruit signaling proteins, tight junctions have also been hypothesized to be involved in the regulation of proliferation, differentiation, and other cellular functions [18,19]. Malignant cells not only accompanied with cell-adhesion abnormity but also frequently display structural and functional disruption of the tight junctions [20]. Recently, the abnormal expression of members of the claudin protein family has been reported to participate in tumorigenesis [21,22]. In particular, claudin-3 and claudin-4 are frequently overexpressed in several neoplasias, including ovarian, breast, pancreatic, and prostate cancers [23]. Moreover, claudin-4 protein is significantly up-regulated in breast invasive ductal carcinomas and is an important correlate with lymphatic metastasis [24]. Although the exact roles of these proteins in tumorgenesis are still being uncovered, it appears that claudin expression has significance during tumor progression [25]. Claudin-5 has been seen in a proportion of gastric carcinomas and seemed to be positively associated with biological markers associated with tumor growth, such as proliferation and apoptosis [26]. It is clear that they represent promising targets for cancer detection, diagnosis, and therapy.

In general, expression of different claudins has not been extensively studied in human tissues and in tumors [27]. An early study in the field showed that occludin was often down-regulated in gastrointestinal tumors [28] and claudin-10 has been found to be reduced in breast cancer as well as in colon cancer [29]. Besides, loss of claudin-17 appears to be associated with a more aggressive behavior of breast carcinoma [30]. These reports of decreased tight junction protein expression in cancer are consistent with the generally accepted idea that tumorigenesis is accompanied by a disruption of tight junctions, a process that may play an important role in the loss of cohesion, invasiveness, and lack of differentiation observed in cancer cells. In addition to the up-regulation or down-regulation of protein levels, phosphorylation of tight junction proteins, including claudins, may affect tight junction function in cancer. Interestingly, phosphorylation of claudin-3 and claudin-4 in ovarian cancer cells has been shown to disrupt tight junctions [31,32]. Paradoxically, other studies have shown that certain claudin proteins are up-regulated in cancer. Overexpression of claudin-3 and -4 has been shown in ovarian carcinoma [33]. In addition, claudin-3 and claudin-4 have also been reported to be expressed in other cancers, such as prostate, and pancreatic cancers [34].

It has been reported that the expression of claudin-2 and claudin-6 was reduced whereas the expression of claudin-11 in gastric cancer was increased in comparison with gastric adjacent non-neoplastic tissues [35]. Claudin-18 gene and claudin-23, frequently down-regulated in intestinal-type gastric cancer and has been shown to have prognostic value in gastric cancer [36]. In summary, in gastric cancer, claudin protein expression has been demonstrated to be of great importance and a relevant area for further study. It is revealed that E-cadherin is specifically required for tight junction formation, but not desmosome, and this appears to involve signaling rather than cell contact formation [37]. Thus, the objective of this study was to examine the expression of claudin-10,-14, -17 and E-cadherin in gastric carcinoma and adjacent tissue which have been less well studied. We used immunohistochemical staining to explore the expression of these proteins in gastric cancer and adjacent non-neoplastic tissues, and correlated the expression of these proteins with tumor differentiation and stage.

## Materials and methods

### Patients

There were 50 cases of gastric cancer and 50 cases of histologically normal adjacent non-neoplastic tissues taken out at more than 2 cm from the tumors were collected from patients being treated at the First Hospital of Jilin University during the period between August 2011 and May 2012. The patients’ medical records were reviewed to determine their age and gender. Sections of the primary tumor were analyzed to identify the histological grade, and the presence or absence of regional lymph node metastasis. There were 34 men and 16 women with average age of 49 years. The cases consisted of 14 well differentiated, 25 moderately differentiated and 11 poor differentiated histological appearance tumors. For the use of these clinical materials for research purposes, prior patient’s consent and approval from the Institute Research Ethics Committee was obtained. All the cancer cases were classified and graded according to the International Union against Cancer (UICC) staging system for gastric cancer.

### Materials

Rabbit monoclonal antihuman E-Cadherin antibody (ab40772), Rabbit polyclonal to claudin-10 antibody (ab66053), Goat polyclonal to claudin-14 antibody (ab115868), Rabbit polyclonal to claudin-17 antibody(ab23333) were purchased from Abcam Technology (USA) and an streptavidin-perosidase immunohistochemistry reagent kit were purchased from Maixin Biology (Fujian, China).

### Criteria for the positive claudin-10,-14 -17 and E-cadherin expression in tissue

The cells positively expressing claudin-10, -14 -17 and E-cadherin were identified by brown staining of cell membrane after reaction with claudin-10, -14,-17 and E-cadherin antibody. The claudin-10,-14,-17 positive tissues were quantified based on the percentage of positive cells which were serially counted in one microscopic field. The cell counting was repeated in five randomly-selected microscopic fields at × 400 magnification. The E-cadherin negative group contained less than 15% positive cells and the positive group, more greater than 15%. The claudin-10 negative groups were defined as a field with level less than 10% (of the tumor cells); positive groups had more than 10% positive cells. The claudin-14 negative group had less than 10% stained cells and the positive group more than 10%. The claudin-17 negative group contained less than 20% positive cells and the positive group, more greater than 20%.

### Statistical analysis

The Chi-square test/Chi-Square Goodness-of-Fit Test was used to determine the prognostic significance value for disease progression of each factor alone, using a *P*-value < 0.05 for statistically significant associations. All the data were analyzed using SPSS 12.0 statistical software.

## Results

### Population and tumor characteristics

The clinicopathological characteristics of the patients are summarized in Table 1.

**Table 1:** Expression of E-cadherin and clinicopathological characteristics in gastric cancer patients

**Item**	**n**	**E-cadherin (+)**	**E-cadherin (-)**	**P**
Gastric cancer tissue	50	16	34	<0.01
Adjacent tissue	50	37	13	
Gender				
Male	34	10	24	0.494*
Female	16	6	10	
Age (year)				
≤60	26	8	18	1.000*
>60	24	8	16	
Histological grade				
Well – differentiated	14	8	6	<0.01
Moderately differentiated	25	7	18	
Poor differentiated	11	1	10	
Lymph node metastasis				
+	24	4	20	<0.01
-	26	12	14	
Ki67				
+	17	6	11	0.356*
-	33	10	23	

^*^No statistical significance.

### The expression of claudin-10, claudin-17 and E-cadherin was reduced in gastric cancer

**Figure 1:**
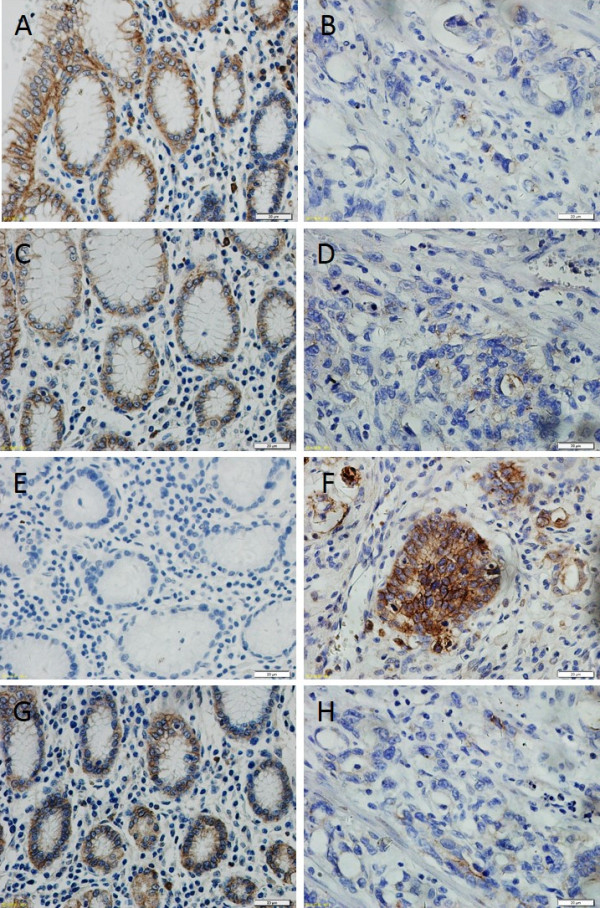
**Immunohistochemical demonstration of claudins protein and E-cadherin expression in human gastric cancer and adjacent tissue.** Claudins and E-cadherin were expressed in the cell membrane. **(A)**, high E-Cadherin expression was detected in tissue adjacent to human gastric cancer compared with low E-Cadherin expression in human gastric cancer tissue **(B)** (400×) **(C)**, claudin-10 was highly expressed in epithelial cells adjacent to gastric cancer but was expressed at low levels in cancer tissue itself **(D)**. **(E)**, the low expression of claudin-14 in tissue adjacent to human gastric cancer compared to strong expression of claudin-14 in human gastric cancer tissue **(F)**. **(G)**, high claudin-17 expression was detected in tissue adjacent to human gastric cancer compared with low claudin-17 expression in human gastric cancer tissue **(H)** (400×).

In our study, E-cadherin expression was evaluated in the membranes of 50 gastric cancers tissues and 50 specimens containing gastric tissue adjacent to the carcinoma. Positive expression of E-cadherin protein was found in 32.0% (16/50) of gastric carcinoma tissues and in 74% (37/50) of adjacent tissues (Table 1). The expression of E-cadherin in gastric cancer tissues was significantly lower than in adjacent tissues (The Chi-square test/Chi-Square Goodness-of-Fit Test, *P* < 0.01) (Figure 1A, B). As shown in Table 1 the expression of E-cadherin was not correlated with age (*P* =1.000), sex (*P* =0.494), expression of Ki 67 (*P* =0.356) but correlated with histological grade (*P* < 0.01) and lymph node metastasis (*P* < 0.01).

Positive expression of claudin-10 protein was found in 24.0% (12/50) of gastric carcinoma tissues and in 72% (36/50) of adjacent tissues (Table 2). The expression of claudin-10 in gastric cancer tissues was significantly lower than in adjacent tissues (The Chi-square test/Chi-Square Goodness-of-Fit Test, *P* < 0.01) (Figure 1C, D). As shown in Table 2 the expression of claudin-10 was not correlated with age (*P* =1.000), sex (*P* =0.664), histological grade (*P* = 1.000), expression of Ki 67(*P* =0.464) but correlated with lymph node metastasis (*P* < 0.01) and expression of E-cadherin (*P* < 0.01).

**Table 2:** **Expression of CLAUDIN-10**, **CLAUDIN-14**, **CLAUDIN-17 and clinic pathological characteristics in gastric cancer patients**

**Item**	**n**	**CLAUDIN-10(+)**	**CLAUDIN-10(-)**	**P**	**n**	**CLAUDIN-14(+)**	**CLAUDIN-14(-)**	**P**	**n**	**CLAUDIN-17(+)**	**CLAUDIN-17(-)**	**P**
Gastric cancer tissue	50	12	38	<0.01	50	29	21	<0.01	50	9	41	<0.01
Adjacent tissue	50	36	14		50	12	38		50	35	15	
Gender												
Male	34	8	28	0.664*	34	20	14	0.355*	34	7	27	0.475*
Female	16	4	12		16	9	7		16	2	14	
Age(year)												
≤60	26	7	19	1.000*	26	15	11	1.000*	26	4	22	0.677*
>60	24	5	19		24	14	10		24	5	19	
Histological grade												
Well –differentiated	14	4	10	1.000*	14	6	8	0.641*	14	3	11	1.000*
Moderately differentiated	25	7	18		25	15	10		25	5	20	
Poor differentiated	11	1	10		11	8	3		11	1	10	
Lymph node metastasis												
+	24	3	21	<0.01	24	18	6	<0.05	24	2	22	<0.05
-	26	9	17		26	11	15		26	7	19	
E-cadherin												
+	16	9	7	<0.0		7	9		16	6	10	<0.01
-		3		1	16	22	12	<0.05	34	3	31	
Ki67			31									
+		5			34	9	8		17	4	13	0.633*
-	34	7	12	0.464*			13			5	28	
			26					1.000*				
	17				17							
	33				33	20			33			

^*^No statistical significance.

Positive expression of claudin-17 protein was found in 18.0% (9/50) of gastric cancer tissues and in 70.0% (35/70) of adjacent tissues (Table 2). The expression rate of claudin-17 in gastric cancer tissues was lower than the rate in adjacent tissues (The Chi-square test/Chi-Square Goodness-of-Fit Test, *P* < 0.01) (Figure 1G, H). As shown in Table 2 the expression of claudin-17 was not correlated with age (*P* =0.677), sex (*P* =0.475), histological grade (*P* = 1.000), expression of Ki 67(*P* =0.633) lymph node metastasis (*P* < 0.05) but correlated with expression of E-cadherin (*P* < 0.01).

### The expression of claudin-14 was increased in gastric cancer

The membrane staining of claudin-14 was strong in gastric cancer tissues and weak in adjacent tissues. Claudin-14 was expressed in 58.0% (29/50) of gastric cancer tissues. Cells were positive for claudin-14 in 24.0% (12/50) of tissues adjacent to the cancer. We conclude that claudin-14 expression is significantly higher (Figure 1E, F) in gastric cancer samples than in histologically normal gastric tissue. (The Chi-square test/Chi-Square Goodness-of-Fit Test, *P* < 0.01). As shown in Table 2 the expression of claudin-17 was not correlated with age (*P* =1.000), sex (*P* =0.355), histological grade (*P* = 0.641), expression of Ki 67 (*P* =1.000) but correlated with lymph node metastasis (*P* < 0.01) and negatively related with expression of E-cadherin (*P* < 0.05).

### Claudin-10, and claudin-14 were concurrently expressed in gastric cancer

We investigated the correlation between claudin-10, claudin-14 and claudin-17 expression using The Chi-square test/Chi-Square Goodness-of-Fit Test. Although we find a correlation between claudin-17 and claudin-10 (The Chi-square test/Chi-Square Goodness-of-Fit Test, φ = 0.693, *P* <0.01) or with claudin-14 (The Chi-square test/Chi-Square Goodness-of-Fit Test, φ = 0.124, *P* = 0.695), we have not find that the expression of claudin-10 correlated with the expression of claudin-14 (The Chi-square test/Chi-Square Goodness-of-Fit Test, φ =0.164, *P* = 0.516). The detailed results of the analysis are described in Table 3.

**Table 3:** Correlation between the expression of claudin-10, claudin-14 and claudin-17

**Item**	**CLAUDIN-10(+)**	**CLAUDIN-10(-)**	φ*	**P**	**CLAUDIN-14(+)**	**CLAUDIN-14(-)**	φ*	**P**
CLAUDIN-17(+)	6	3	0.693	<0.01	5	4	0.124	0.695
CLAUDIN-17(-)	6	35			24	17		
CLAUDIN-14(+)								
	7	22	0.164	0.516				
CLAUDIN-5		16						
14(-)								

*φ Phi coefficient.

## Discussion

The majority of human cancers originate from epithelial cells. Normally, epithelial cells are tightly interconnected through several junction structures, including tight junctions, adherents-type junctions and desmosomes, which are intimately associated with the actin and intermediate cytoskeleton. Including carcinomas of the breast, colon, prostate, liver, skin, kidney and lung appear to cause the loss of E-cadherin function, caused by several different mechanisms including deletion or mutational inactivation of the E-cadherin gene [38]. Notably, mutations in the E-cadherin gene are evident in cases of familial gastric cancers, which indicates that mutation of the E-cadherin gene is sufficient to predispose individuals to the development of malignant cancer. Moreover, the loss of E-cadherin impair E-cadherin-mediated cell-cell adhesion and E-cadherin contributes to epidermal barrier formation by regulating the incorporation of claudins into tight junctions and is specifically required for correct tight junction formation [39].

Although the normal ratio of claudin proteins has a role in maintaining the structure and function of tight junctions in epithelial cells, the mechanisms by which claudins expression and destruction of tight junctions induce tumor formation and the effect of these changes on tumor progression have not been studied in detail [40]. It has been postulated that both abnormal expression and phosphorylation of claudin proteins would cause the structural and functional disruption of tight junctions [41]. Currently, it is reported that the alternation of claudins expression is one of the mechanisms responsible for loss of cell adhesion, altered polarity, poor differentiation and increased invasive potential of neoplastic cells [42]. In this study, expression of claudin-10, 14, 17 and was studied in 50 cases of gastric carcinoma and adjacent non-neoplastic tissues adjacent to the gastric carcinoma tissues. The results show variable and heterogenous expression of claudin-10, -14 and-17 in gastric carcinoma. The most prominent expression of claudins was seen for claudins-14, where about 58% of cases showed positivity, whereas expression was weaker for claudin-10, which showed 24% of cases positive, and for claudin-17, which showed 18% of cases positive. In accordance with this, we also observed lower E-cadherin expression in diffuse carcinomas. It might also be that loss of claudins and E-cadherin expression is somehow interrelated in as much as E-cadherin has been shown to influence the formation of tight junctions and desmosomes although it mainly mediates the assembly of adherents junctions. In line with this, there was an association between expression of claudins-4 and -5 and E-cadherin [43]. However, little data are available on the functional association between E-cadherin and claudins at present. Several proteins were identified that were associated with prognosis of the gastric cancer patients, for instance, It is reported that Astragalus saponins inhibited human gastric cancer cell growth, decreased the invasion ability and induced the apoptosis and Variable copy number of mitochondrial DNA (mtDNA) predicts worse prognosis in advanced gastric cancer patients [44,45]. In addition to this, inducible nitric oxide synthase expression in gastric adenocarcinoma related with lymph angiogenesis and lymphatic metastasis and the expression of TIMP3 gene may provide evidence for the molecular diagnosis and stage evaluation of gastric cancer [46,47]. Our data revealed that E-cadherin expression has been associated with a poorer prognosis of the patients. In general, such an association could not be seen with claudins, except for claudin-3, where its lowered expression was associated with a marginally poorer prognosis [48].

In conclusion, our present data observes tight junction proteins claudins-10, -14, -17 and E-cadherin between human gastric cancers and adjacent non-neoplastic tissues correlate with lymph node metastasis. In addition, claudin-10, claudin-17 and E-cadherin. In this respect, the loss of the claudin-10 and claudin-17 may resemble E-cadherin and together with this molecule, might contribute to the loose cell cohesion in gastric cancer.

## Conclusion

The present work infers that the expression altered of claudin-10, claudin-14, claudin-17 and E-cadherin between human gastric cancers and adjacent non-neoplastic tissues correlate with lymph node metastasis. In addition, claudin-10, claudin-17 and E-cadherin were concurrently expressed in gastric cancer. However, the specific mechanism responsible for these observations needs to be addressed in the future.

## Competing interests

The authors declare that they have no competing interests.

## Authors’ contributions

GW carried out part of experiments, participated in the design of the study, performed the statistical analysis, and drafted the manuscript. MG and WL carried out most of experiments, and helped draft the manuscript. HW assisted with the experiments, and helped to edit the paper. All authors have read and approved the final manuscript.
